# Electroencephalographic characteristics of transcutaneous auricular vagus nerve stimulation for prolonged disorders of consciousness: a study protocol

**DOI:** 10.3389/fnins.2025.1539232

**Published:** 2025-05-06

**Authors:** Haoyang Jiao, Weihang Zhai, Jinling Zhang, Long Xu, Xiaoli Geng, Xueling Chen, Yi Yang, Yifei Wang

**Affiliations:** ^1^Institute of the History of Chinese Medicine and Literature, China Academy of Chinese Medical Sciences, Beijing, China; ^2^Institute of Basic Research in Clinical Medicine, China Academy of Chinese Medical Sciences, Beijing, China; ^3^Institute of Acupuncture and Moxibustion, China Academy of Chinese Medical Sciences, Beijing, China; ^4^Department of Neurosurgery, Capital Medical University Affiliated Beijing Tiantan Hospital, Beijing, China

**Keywords:** prolonged disorders of consciousness, transcutaneous auricular vagus nerve stimulation, electroencephalographic characteristics, power spectrum, functional connectivity

## Abstract

**Background:**

Increasing evidence indicates that transcutaneous auricular vagus nerve stimulation (taVNS) can promote the recovery of consciousness levels in patients with prolonged disorders of consciousness (pDOC). However, previous clinical studies have shown inconsistent clinical efficacy of taVNS in treating pDOC. This could be because no research has clearly defined the indications for taVNS treatment in patients with pDOC, and patients who were not indicated for taVNS treatment were included. Therefore, screening out the indications for patients with pDOC who are suitable for taVNS treatment is a key scientific issue that requires urgent resolution at present.

**Methods/design:**

We aim to incorporate 50 patients with pDOC into this study. All patients will undergo a 4-week taVNS treatment, and 30-min resting electroencephalogram (EEG) and Coma Recovery Scale-Revised (CRS-R) scores of each patient will be collected before and after the treatment. After the 4-week taVNS treatment, the patients will be followed up in the first, third, sixth, and twelfth months after the treatment. The 50 patients with pDOC will be classified into response and non-response groups based on whether their CRS-R scores increase. The differences in the brain power spectrum and functional connectivity of the EEG before enrollment between the two groups will be compared.

**Discussion:**

The research objective of this experiment is to screen out the EEG characteristics of patients with pDOC who are suitable for taVNS treatment by using EEG technology, and lay the foundation for the wider application of taVNS in the treatment of patients with pDOC.

**Clinical trial registration:**

Identifier ITMCTR2024000734, http://itmctr.ccebtcm.org.cn/.

## Introduction

The disruption of cortical-thalamic and cortical–cortical connections in pDOC is primarily attributed to extensive damage to long-range white matter fiber tracts following severe brain injury (Laouchedi et al., 2015). Currently, neuroregulation represents the main therapeutic approach. However, invasive neuroregulatory techniques often deter many patients and their families due to high costs and perioperative risks.

Vagus nerve stimulation therapy is used to regulate brain activity through electrical stimulation of the vagus nerve (Badran et al., 2022). The distribution of the vagus nerve in the ear branch is mainly concentrated in the auricular region, including the cymba conchae and the cavitas conchae (Borges et al., 2021). Therefore, electrical stimulation of the auricular branch of the vagus nerve can produce similar effects to classical vagus nerve stimulation without perioperative risks (Sarma et al., 2023). Several previous findings have shown that vagus nerve stimulation modulates or activates cortical and subcortical areas associated with conscious control, including the cortical lobules, nucleus ambiguus, hypothalamus, medial prefrontal cortex, dorsolateral prefrontal cortex, anterior cingulate cortex, and posterior cingulate cortex (Zhi et al., 2024; Jang and Cho, 2022). taVNS is increasingly favored by patients’ families and clinicians because of its simplicity, accessibility, inexpensiveness, and effectiveness.

Currently, the level of consciousness in patients with pDOC is clinically differentiated by using the CRS-R, as it is a good discriminator between vegetative state/unresponsive wakefulness syndrome (VS/UWS) and minimally conscious state (MCS) (Annen et al., 2019). Relevant studies have pointed out that the misdiagnosis rate of pDOC patients using the CRS-R is as high as 40% (Wang et al., 2020). Clinically, functional magnetic resonance imaging (fMRI) is often used to improve the accuracy of detecting the conscious state of patients with pDOC. However, due to its high cost, contraindications for fMRI examinations such as metal implants, and the possible involuntary head movements of patients, which can generate significant artifacts in fMRI and interfere with the accurate acquisition and analysis of brain signals, the test results may deviate (Smith et al., 2021). In recent years, EEG techniques have been gradually applied to clinical consciousness assessment because of their low price, convenient use at the bedside, and high diagnostic accuracy, and EEG is especially suitable for evaluating noninvasive neuromodulation techniques at the bedside for brain network intervention in patients with pDOC because of its high temporal resolution and simple operation (Lo et al., 2024; Liuzzi et al., 2022).

Several studies have confirmed the efficacy and safety of taVNS in promoting arousal in patients with pDOC. A 6-month case study of taVNS for treating pDOC was carried out. During the daily taVNS treatment of the patient, the CRS-R was used to regularly evaluate the patient’s behavioral indicators. Meanwhile, electrophysiological data such as resting-state EEG, heart rate (HR), and heart rate variability (HRV) were collected. The results showed that although the total CRS-R score of the patient fluctuated, it gradually increased from 4–6 points in the initial stage to a peak of 12–13 points in the 3rd and 5th months, and the patient’s conscious state changed from VS to MCS (Osińska et al., 2022). In a previous study of ours, 12 patients with pDOC were treated with taVNS for 2 weeks. After the treatment, none of the 12 patients showed an improvement in the CRS - R score, and only changes in EEG functional connectivity were observed (Yifei et al., 2022). However, the efficacy varies significantly between studies, which may be related to the fact that no study has yet clarified the indications of taVNS for the treatment of patients with pDOC, and patients with pDOC who are not indications for taVNS treatment were included. Therefore, identifying the indications of taVNS for the treatment of pDOC is a key scientific issue that needs to be addressed.

In this trial, we attempted to reveal the EEG characteristics of pDOC treated with taVNS using EEG technology. For pDOC, which is a special intractable disease, it is very important at both social and economic levels to identify the patient population suitable for taVNS treatment through screening at the early stage of the disease. Such a screening strategy can help optimize the allocation of medical resources, improve treatment efficiency, reduce unnecessary medical expenditures, and provide patients with more precise and personalized treatment plans.

## Methods

### Study design

This study is a prospective, exploratory clinical trial that has completed registration in the International Traditional Medicine Clinical Trial Registry (ITMCTR2024000734). The study protocol was designed in accordance with the Declaration of Helsinki and approved by the Ethics Committee of the Institute of Acupuncture and Moxibustion, China Academy of Chinese Medical Sciences. As the patients in this trial were incapacitated, the informed consent was signed by the patients’ legal representatives.

### Participants

This study will screen out 50 patients with pDOC through strict inclusion and exclusion criteria.

#### Inclusion criteria

(1) Diagnosed with pDOC based on the Coma Recovery Scale-Revised; (2) Age 18 to 70; (3) Illness duration of more than 1 month; (4) Normal cardiac, pulmonary, hepatic, and renal function; (5) Expected survival time is greater than 3 months; (6) The patient’s legal representative agrees to sign a written informed consent form; (7) The female patient’s pregnancy test is negative; (8) Coagulation function is normal.

#### Exclusion criteria

(1) Neurodegenerative diseases, intracranial infections, and post-craniotomy coma due to brain tumors; (2) Continuous improvement or deterioration of consciousness within 1 month prior to enrollment; (3) Severe complications; (4) Participated in other clinical trials within 3 months prior to the study; (5) History of severe allergies or allergic constitution; (6) Severe medical illnesses or severe uncontrolled infections; (7) Drug abuse, substance misuse, chronic alcoholism, and HIV/AIDS.

#### Withdrawal criteria

(1) Recurrent seizures during the treatment period; (2) Death; (3) Patient is lost to follow-up.

The inclusion criteria, exclusion criteria, withdrawal criteria are presented in Table 1.

**Table 1:** Include, exclude and withdrawal criteria.

Inclusion
1. Diagnosed with DOC based on the Coma Recovery Scale-Revised;
2. Age 18 to 70;
3. Illness duration of more than 1 month;
4. Normal cardiac, pulmonary, hepatic, and renal function;
5. Expected survival time is greater than 3 months;
6. The patient’s legal representative agrees to sign a written informed consent form;
7. The female patient’s pregnancy test is negative;
8. Coagulation function is normal.

Exclusion
1. Neurodegenerative diseases, intracranial infections, and post-craniotomy coma due to brain tumors;
2. Continuous improvement or deterioration of consciousness within 1 month prior to enrollment;
3. Severe complications;
4. Participated in other clinical trials within 3 months prior to the study;
5. History of severe allergies or allergic constitution;
6. Severe medical illnesses or severe uncontrolled infections;
7. Drug abuse, substance misuse, chronic alcoholism, and HIV/AIDS.

Withdrawal
1. Recurrent seizures during the treatment period;
2. Death;
3. Patient is lost to follow-up.

### Procedures

We plan to enroll 50 patients with pDOC in this study. Each patient will receive 4 weeks of taVNS treatment, and we will collect 30-min resting EEG and CRS-R scores before and after 4 weeks treatment. In addition to taVNS, all patients received daily foundational therapy primarily comprising rehabilitation training. The EEG will be taken by an experienced technician, We will follow up the patients at the first, third, sixth and twelfth month after 4 weeks of taVNS treatment. Due to patients’ limited mobility, follow-up assessments were conducted Tencent Meeting and WeChat groups. Two neurosurgeons certified in standardized CRS-R administration performed independent evaluations. Discrepancies in ratings were adjudicated by a senior attending physician. To ensure the reproducibility and accuracy of results, follow-up evaluations were conducted daily between 9:00 AM and 11:00 AM in a controlled environment where patients had not received sedative medications prior to assessment.

We categorized 50 patients into response and non-response groups based on whether or not their CRS-R scores increased. To address inter-group baseline differences, we employed analysis of covariance with baseline CRS-R scores as covariates to compare pre-intervention EEG differences between groups in terms of power spectrum and functional connectivity, aiming to identify EEG biomarkers characterizing pDOC patients eligible for taVNS treatment.

**Figure 1:**
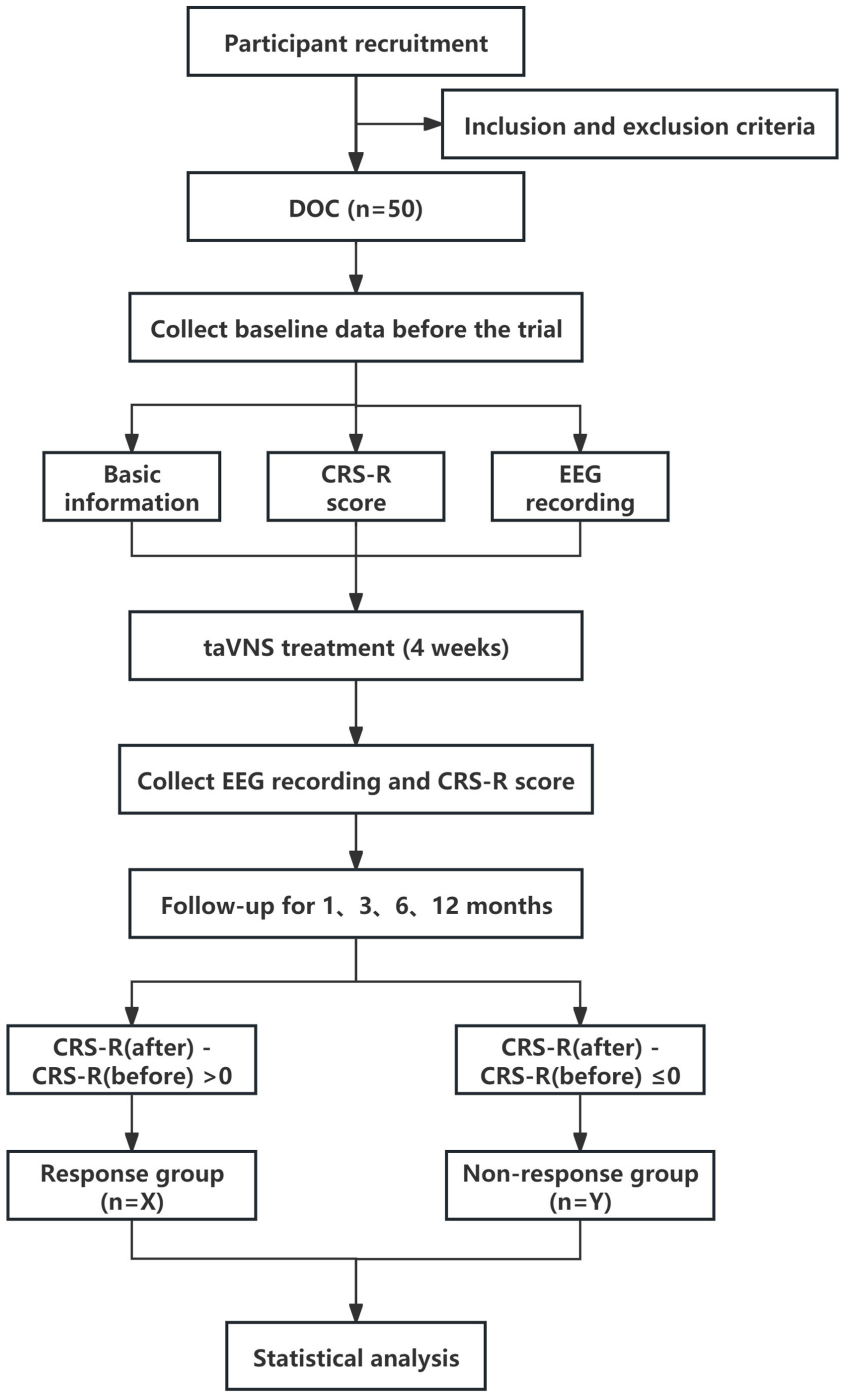
Study flowchart. pDOC, prolonged disorders of consciousness; CRS-R, Coma Recovery Scale-Revised; taVNS, transcutaneous auricular vagus nerve stimulation; EEG, Electroencephalogram.

During the experiment, the Ethics Committee of the Institute of Acupuncture and Moxibustion of the China Academy of Chinese Medical Sciences will be responsible for independent safety monitoring. Adverse events and unexpected events will be reported to them when every 10 patients are included. They will further assess the causal relationship between adverse events and treatment. If the treatment proves to have caused a serious adverse event, the trial will be terminated immediately. Appropriate medical emergencies and protective measures will also be provided to the patients (Figure 1).

### Stimulations

**Figure 2:**
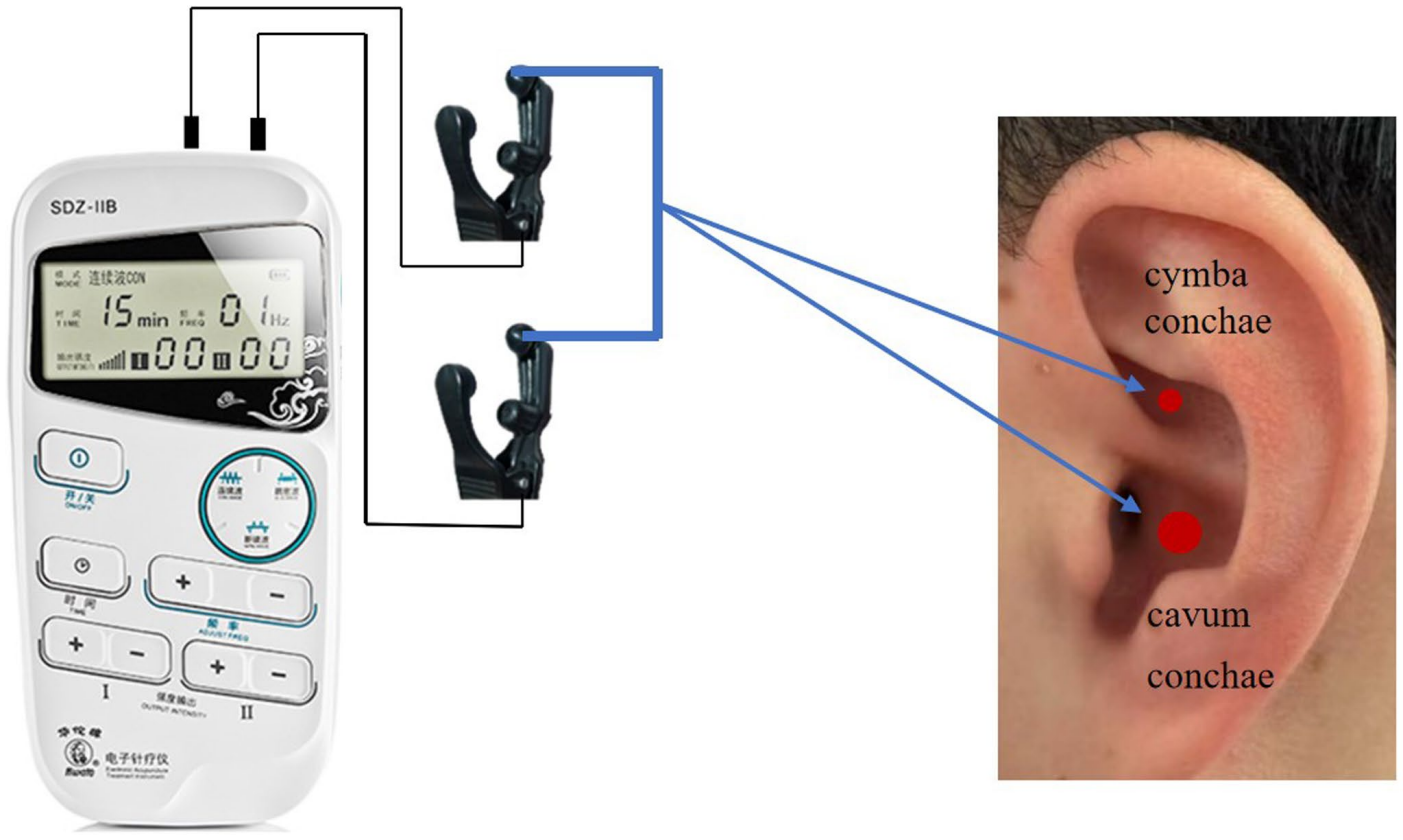
Stimulation sites of taVNS.

**Figure 3:**
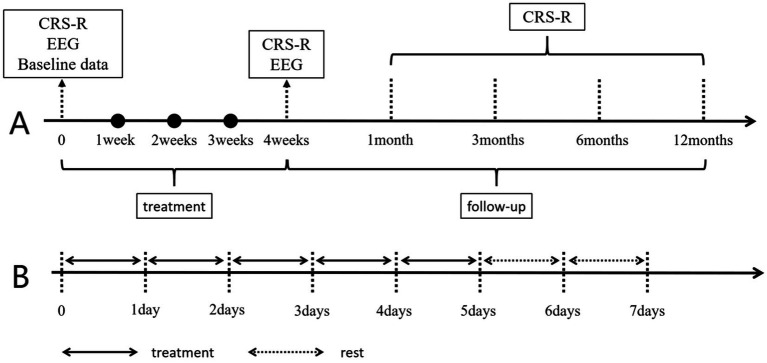
Treatment and data collection time. Panel **A** shows the data collection time, and Panel **B** shows the weekly taVNS treatment and rest conditions. CRS-R, Coma Recovery Scale-Revised; EEG, Electroencephalogram.

The specific manipulation of taVNS is as follows: For taVNS (SDZ-IIB, Suzhou Medical Supplies Factory), the stimulation site is the auricular concha area of the ear, mainly the cymba conchae and cavum conchae. A pair of clips with identical appearance were placed on both ears. The clips were designed with three carbon-impregnated silicone tips. One of them served as the common terminal for supporting the posterior surface of the auricle, and the other two tips were designed to stimulate two skin surface points, one on the outer ear and the other on the scaphoid. The two silicone tips at the outer ear points were placed, respectively, in the cavum conchae and the cymba conchae, as shown in Figure 2. Each patient wore a heart rate monitoring device during the research period. All enrolled patients received taVNS treatment in addition to the basic treatment, targeting the “heart” and “kidney” acupoints on the ear, with a dense-sparse wave, a frequency of 4/20 Hz, and an intensity appropriate such that the patient did not exhibit obvious heart rate fluctuations, with a current of approximately 1–1.5 mA. Each treatment lasted for 30 min, with two treatments per day, one in the morning and one in the evening, with 5 days of treatment followed by 2 days of rest, for a total of 4 weeks of treatment. After the 4-week taVNS treatment, we will conduct follow-ups on the patients in the first month, third month, sixth month, and twelfth month after the treatment, as shown in Figure 3.

### Behavioral assessments

The CRS-R score is a clinical rating scale employed for assessing the recovery conditions of patients with pDOC, particularly applicable for the differential diagnosis of patients in a VS/UWS and MCS. The CRS-R rating scale is based on the patient’s response ability to external stimuli, evaluating the recovery degree of their consciousness, response, and cognition. The scale totals 23 points and encompasses six subscales: auditory, visual, motor, language, communication, and arousal level. In the research, the CRS-R score was rated by two experienced physicians. In case of disputes in the ratings given by the two physicians, it was reported to a higher-level physician for adjudication. We conducted the scoring, respectively, before treatment, after treatment, and at weekly time points during the treatment process, and conducted follow-up scoring at the 1st, 3rd, 6th, and 12th months after treatment. Due to patients’ limited mobility, follow-up assessments were conducted Tencent Meeting and WeChat groups. Two neurosurgeons certified in standardized CRS-R administration performed independent evaluations. Discrepancies in ratings were adjudicated by a senior attending physician. To ensure the reproducibility and accuracy of results, follow-up evaluations were conducted daily between 9:00 AM and 11:00 AM in a controlled environment where patients had not received sedative medications prior to assessment. A total of nine CRS-R assessments were completed per participant across all predefined time points.

### EEG recording and analysis

Before and after 4 weeks of taVNS treatment, we recorded 30 min of resting EEG data from each patient. The EEG will be acquired at a sampling rate of 1,000 Hz through 32 channels (Nicolet EEG V32, Natus Neurology, USA), and the impedance between the electrodes and the patient’s skin will be maintained at sub-5 kΩ at all times. During EEG recordings, the patient will remain awake and if they show signs of sleepiness, the subject will be awakened, otherwise the recording will be suspended.

#### Preprocessing

The raw data will be imported into the data analysis software Matlab 2016b and EEGlab, the channel position information matching the time of recording the data will be loaded to determine the position of each electrode on the scalp, the data segments with large artifacts will be visually removed, the bandpass filtering will be set to 1-70 Hz, the sampling rate will be reduced to 500 Hz, and the components of the ocular and noise will be excluded by ICA, and the resting The resting EEG data were segmented into 10s segments for storage and subsequent study.

#### Relative power calculation

The total power of the six frequency bands delta (1–4 Hz), theta (4–8 Hz), alpha (8–13 Hz), beta (13–30 Hz), low gamma (30–47 Hz), and high gamma (52–70 Hz) was calculated, and the ratio of each band to the total band was used as the relative power of the band, which was calculated by the following formula. The formula is as follows.


$$\mathrm{powe}r_{r}\left(f_{1}\;f_{2}\right)=\frac{\mathrm{power}\left(f_{1},f_{2}\right)}{\mathrm{power}\left(1,70\right)}\times 100\%$$

$\mathrm{powe}r_{r}\left(f_{1}\;f_{2}\right)$ Indicates the total energy between frequencies *f1* and *f2*, and $\mathrm{power}\left(1,70\right)$ indicates the total energy from 1 to 70 Hz.

The functional connectivity index selected in this study is the Weighted Phase-Lag Index (wPLI), which is used to assess the phase synchronization between different channels or regions in EEG signals. wPLI mainly focuses on the instantaneous phase relationship between the signals, especially the phase lag or overshooting, to reflect the strength of information exchange or functional connectivity between different brain regions. The calculation of wPLI is based on the imaginary portion of the cross-spectrum between two signals and is weighted to enhance the detection of true phase synchronization. Compared with conventional phase synchronization metrics, wPLI is more robust to volumetric conduction effects and the choice of reference electrodes, and therefore more accurately reflects the true synchronization of neural activity.

Briefly, the calculation process of wPLI includes the following steps: (1) Calculate the cross-spectrum between two signals: for a given two signals, first calculate their Fourier transforms, and then obtain the cross-spectrum. (2) Extract the imaginary component of the cross-spectrum. Since wPLI is concerned with phase synchronization, it is necessary to extract the imaginary component from the cross-spectrum, which contains information about the phase difference between the two signals. (3) Calculate the phase lag index: the phase lag index is a synchronization metric based on the phase difference, which ignores the cases where the phase difference is 0 or *π* in order to avoid being affected by the volumetric conduction effect. (4) Weighting process to get wPLI. By weighting process, wPLI can reflect the real phase synchronization between two signals more accurately.

### Data safety and management

The medical histories, demographic data, behavioral data, and EEG data of all patients will be stored by the study director in the computer database of the department. The Ethics Committee of the Institute of Acupuncture and Moxibustion of the China Academy of Chinese Medical Sciences will periodically inspect the data security.

### Statistical analysis

Statistical analysis was performed using IBM SPSS26 software. Measurement data such as age, disease duration, and CRS-R score conforming to normal distribution were expressed as mean standard deviation, and count data such as gender and etiology were applied to chi-square analysis. Repeated-measures ANOVA was used to compare the changes in EEG power spectrum indexes before and after treatment in the two groups of patients. A two-sample *t*-test was performed on the EEG functional connectivity connecting edges with FDR correction. The statistical significance level was set at *p* < 0.05.

## Discussion

Related studies have indicated that taVNS is feasible and safe for the treatment of patients with pDOC. In 2017, our team reported the world’s first case of taVNS awakening a pDOC patient (Yu et al., 2017). A 73-year-old female patient, after undergoing taVNS treatment for 4 weeks, her CRS-R score increased from 6 to 13, and her consciousness state recovered from VS/UWS to MCS.

In 2021, our team reported the influence of taVNS on cerebral hemodynamics in pDOC patients (Yu et al., 2021). This study included a total of 10 pDOC patients, with 5 responding to auditory stimulation and 5 not responding. After 4 weeks of taVNS treatment, the patients responding to auditory stimulation showed increased cerebral blood flow in multiple brain regions, a significant improvement in the CRS-R score, and a good prognosis as indicated by the Glasgow Outcome Scale (GOS). In contrast, for the patients not responding to auditory stimulation, the increase in cerebral blood flow after taVNS treatment was relatively weak, with only a significant increase in cerebral blood flow in the left cerebellum. The CRS-R score and GOS score did not show significant changes. Therefore, the preservation of auditory function may be a prerequisite for the effectiveness of taVNS in treating pDOC patients.

In 2022, a small sample study by our team found that after 2 weeks of taVNS treatment for 12 pDOC patients, the EEG results showed that in MCS patients, the energy in the delta band decreased and the energy in the beta band increased, while the opposite was true for VS/UWS patients (Yifei et al., 2022).

Hakon et al. (2020) treated 5 patients diagnosed as VS/UWS or MCS with taVNS for 8 weeks. The results revealed that 3 out of 5 patients showed improved consciousness. Specifically, 2 patients progressed from VS/UWS and MCS to Emerging from the minimally conscious state (EMCS), and 1 patient progressed from VS to MCS. In another study, 14 pDOC patients (VS = 6, MCS = 8) received taVNS treatment in the left ear for only 4 weeks. One MCS patient showed new signs of consciousness at the end of 4 weeks of stimulation, and another 4 patients showed new signs of consciousness at the 4-week follow-up time point (Noé et al., 2020).

In terms of EEG studies. Some studies have shown that enhanced delta activity and suppressed alpha activity are prominent markers of a low level of consciousness (Rossi Sebastiano et al., 2015). The suppression of alpha power in VS/UWS patients is much stronger than that in MCS patients (Casarotto et al., 2024), and the alpha power of MCS patients is significantly higher than that of VS/UWS patients (Wei et al., 2023; He et al., 2023), sometimes even up to twice (Naro et al., 2018). Compared with VS/UWS patients, MCS patients have higher alpha power in the central, parietal, and occipital regions (Rossi Sebastiano et al., 2015). The residual consciousness of pDOC patients is closely related to the alpha power of the frontal and parietal networks (Coulborn et al., 2021; Liu et al., 2023). Additionally, the increase in alpha band energy over time is associated with consciousness recovery (Zhou et al., 2024; Han et al., 2022). Related studies have indicated that the higher the consciousness level of pDOC patients, the stronger the functional connectivity between various brain regions (Chen et al., 2022; Altmayer et al., 2024). The prefrontal cortex plays a crucial role in the formation of consciousness (Panagiotaropoulos TI, 2024; Pal et al., 2018). Compared with normal individuals, the information interaction between the frontal–parietal and frontal-temporal regions in pDOC patients is significantly weakened (Liu et al., 2023). A recent study by Panda et al. (2022) found that the occurrence of prolonged disorders of consciousness is related to the disruption of effective connectivity between the temporal and parietal regions of the cerebral cortex.

We have discovered that the clinical efficacies of different taVNS treatments for pDOC patients in clinical studies vary significantly. Therefore, identifying the indications for taVNS treatment of pDOC patients is a scientific problem that urgently needs to be addressed. Resting-state EEG is widely applied in clinical settings as subjects do not need to perform any tasks and there is no stimulation. In this trial, we attempt to utilize EEG technology to screen for the EEG characteristics of pDOC patients suitable for receiving taVNS treatment. For this particular and refractory disease, identifying the patient group suitable for receiving taVNS treatment in the early stage of the disease is of great significance at both the social and economic levels. Such a screening strategy helps optimize the allocation of medical resources, improve treatment efficiency, reduce unnecessary medical expenditures, and provide more precise and personalized treatment plans for patients.
